# Overexpression of *S30* Ribosomal Protein Leads to Transcriptional and Metabolic Changes That Affect Plant Development and Responses to Stress

**DOI:** 10.3390/biom14030319

**Published:** 2024-03-07

**Authors:** Alin Finkelshtein, Hala Khamesa-Israelov, Daniel A. Chamovitz

**Affiliations:** 1 School of Plant Sciences and Food Security, Tel Aviv University, Ramat Aviv 69978, Israel; 2 The Department of Life Sciences, Ben-Gurion University of the Negev, Be’er Sheva 84105, Israel

**Keywords:** ribosome, indole-3-carbinol, metabolomics, photomorphogenesis, plant development, stress resistance

## Abstract

ICT1 is an *Arabidopsis thaliana* line that overexpresses the gene encoding the S30 ribosomal subunit, leading to tolerance to exogenous indole-3-carbinol. Indole-3-carbinol (I3C) is a protective chemical formed as a breakdown of I3M in cruciferous vegetables. The overexpression of S30 in ICT1 results in transcriptional changes that prime the plant for the I3C, or biotic insult. Emerging evidence suggests that ribosomal proteins play important extra-ribosomal roles in various biochemical and developmental processes, such as transcription and stress resistance. In an attempt to elucidate the mechanism leading to I3C and stress resistance in ICT1, and using a multi-pronged approach employing transcriptomics, metabolomics, phenomics, and physiological studies, we show that overexpression of S30 leads to specific transcriptional alterations, which lead to both changes in metabolites connected to biotic and oxidative stress tolerance and, surprisingly, to photomorphogenesis.

## 1. Introduction

ICT1 is an *Arabidopsis thaliana* line that overexpresses the gene encoding the *S30* ribosomal subunit, leading to tolerance to exogenous indole-3-carbinol (I3C) [1]. I3C is formed from the breakdown of indole-3-methylglucosinolate (I3M), the predominant indole glucosinolate [2], by a reaction primarily catalyzed by myrosinase [3,4], though the formation of I3M can also occur in the absence of myrosinase [5]. In Arabidopsis, I3C serves as a signaling molecule that modulates the auxin response by antagonizing auxin binding to the TIR1 receptor and other mechanisms [6,7,8]. Hence, exogenous I3C at high concentrations inhibits *Arabidopsis* growth and development [7].

While ICT1 was specifically tolerant to I3C and not to other toxic molecules, it was also tolerant to bacterial and fungal pathogens, as well as to oxidative stress [1]. The overexpression of *S30* in ICT1 results in transcriptional changes that prime the plant for the I3C, or biotic insult. For example, the genes involved in responses to biotic stress, immune responses, and stress responses are expressed constitutively, priming the plant to respond more quickly [1].

The ribosome, a two-subunit ribonucleoprotein complex, plays a pivotal role in protein synthesis but also has emerging roles in transcriptional regulation. In *Arabidopsis thaliana*, the large 60S subunit consists of 48 ribosomal proteins (RPs), while the small 40S subunit contains 32 RPs, including *S30* [9]. *S30* is required for ribosome assembly and protein synthesis by promoting the interaction between the tRNA anticodon stem-loop and the 18S rRNA 530 loop [10].

Mounting evidence indicates that differences in RP expression can impact functions unrelated to ribosome assembly or protein synthesis. For example, mutations and differential expression in ribosomal genes have been found to play a role in regulating normal development and responding to environmental stresses [11,12,13,14]. In an attempt to elucidate the mechanism leading to I3C and stress resistance in ICT1, and using a multi-pronged approach employing transcriptomics, metabolomics, phenomics, and physiological studies, we show that overexpression of *S30* leads to specific transcriptional alterations, which lead to both changes in metabolites connected to biotic and oxidative stress tolerance and, surprisingly, to photomorphogenesis.

## 2. Materials and Methods

### 2.1. Plant Material and Growth Assays

The *Arabidopsis* lines used in this work were all WT ecotype Columbia-0 backgrounds. Transgenic and mutant lines were previously reported: ICT1 [1], *S30*^t-DNA^ (Salk_087911.4) [15], *tgg1/2* double mutant [16], and *cyp79B2/B3* double mutant [17]. Seeds were cultivated in Petri plates using medium containing 0.8% agar (Acumedia, Lansing, MI, USA, 7178A), half-strength Murashige and Skoog salts (MS) (Sigma, Yokohama, Japan, M5519), and 1% sucrose (Bio Lab, Rockville, MD, USA, 19220591, pH 5.7. The Petri plates were placed in chambers at 21 °C under light/dark conditions of 16 h white light at 75 lmol m^2^ sec^−1^ and 8 h darkness, at 55% relative humidity. For root phenotype experiments, plates were placed vertically in the chambers. All treatments were conducted under the same day-time conditions. For experiments that included ICT1, at least 3 independent lines of ICT1 were used in each; the results are the average of all lines.

*Hydroponic growth:* For treatments with I3C, plants were grown hydroponically in tall petri dishes with liquid MS. ~60 seedlings were in each plate for two weeks in normal conditions (long day, 21 °C, humidity 55%). All lines were exposed for two hours to either 0.05% DMSO or 400 µM I3C (diluted in 0.05% DMSO). All samples were collected at the same time to eliminate circadian clock effects and washed three times with fresh DDW. Moreover, 10 seedlings for each sample were pooled, frozen in liquid nitrogen, and lyophilized. Indole-3-carbinol (I3C) (Sigma I7256)was dissolved in 0.05% DMSO (Merck, Rahway, NJ, USA, 1.02931) to produce a 1 M solution and stored in the dark at −20 °C.

### 2.2. Yield

ICT1 and WT lines were grown in pots in optimal conditions until full ripening. Seeds were collected separately from each plant and weighted. The weight was transformed into the number of seeds with the aid of a calibration curve. The average weight and number of seeds per plant were calculated.

### 2.3. LC-MS-MS

*Sample preparation:* Seedlings were grown hydroponically. Dry samples were powdered with 2 stainless-steel balls in a beat beater (1 min, MM400, Retsch, Haan, Germany). One mg of each sample was extracted with 80% methanol (Bio Lab, 1368060200) in 0.2% formic acid (500 uL), containing ethylparaben (1 ng/mL) as an internal standard (IS), by shaking (20,000 rpm, 65 °C, 15 min, Thermomixer C, Eppendorf, Hamburg, Germany). Then, the mixtures were centrifuged (14,000× *g*, 10 min), and supernatants were collected and placed in LC-MS vials. Standard curve mixtures of 0.001–10 ug/mL of I3C and DIM were prepared in 80% methanol/0.2% formic acid, containing 1 ug/mL of ethylparaben as IS.

*Standards:* Indole-3-carbinol (I3C) I7256, 3,3′-diindolylmethane (DIM) D9568, and ethylparaben PHR1011 (internal standard, IS) were purchased from Sigma/Merck.

LC-MS-MS analysis was performed on the Acquity UPLC system and the triple quadrupole Xevo TQ-S (both Waters). TargetLynx (Waters, Milford, MA, USA) was applied for quantitation based on standard curves.

*LC parameters*: Acquity BEH C18 column (2.1 × 100 mm, 1.7 µm; Waters) at 35 °C. Gradient with mobile phases A—0.01% aqueous formic acid and B—0.01% formic acid in acetonitrile: 0 min, 20%B; 3 min, 100%B; 4 min, 100%B; 4.5 min, 20%B; 7 min, 20% B. Injection volume: 2.0 µL; flow rate: 0.3 mL/min. Retention times: 2.25 min for I3C, 2.96 min for DIM, and 2.18 min for IS.

*MS/MS parameters*: Electrospray ionization in positive-ion mode; desolvation temperature, 400 °C; desolvation gas flow, 600 L/hr; cone gas flow, 150 L/hr; nebulizer pressure, 7 Bar; capillary voltage, 3.56 kV; cone voltage, 44 V; with argon 0.10 mg/min as the collision gas. The MRM transitions are: 130.1 > 77.0 and 130.1 > 103.1 with collision energy (CE) 22 V in both I3C and DIM; 167.1 > 95.0 and 167.1 > 139.1, with CE 13 and 10 V, respectively, for IS.

### 2.4. LC-MS

Sample preparation: seedlings were grown in MS plates, with ~60 seeds in each plate, vertically standing for 16 days in normal conditions.

With and without I3C treatment, ICT1 and WT were grown hydroponically. Each tube contains 100 mg of wet weight.

Metabolic profiling of the semipolar phase was performed using the Waters ACQUITY UPLC system coupled to a Vion IMS QTof mass spectrometer (Waters Corp., MA, USA). The chromatographic separation was performed on an ACQUITY UPLC BEH C18 column (2.1 × 100 mm, i.d., 1.7 μm) (Waters Corp., MA, USA). The mobile phase A consisted of 95% water (UPLC grade) and 5% acetonitrile, with 0.1% formic acid; the mobile phase B consisted of 100% acetonitrile with 0.1% formic acid. The column was maintained at 35 °C, and the flow rate of the mobile phase was 0.3 mL*min^−1^. Mobile phase A was initially run at 100%, and it was gradually reduced to 72% at 22 min, following a decrease to 60% at 22.5 min and 0% at 23 min. Then, mobile phase B was run at 100% until 26.5 min, and mobile phase A was set to 100% at 27 min. Finally, the column was equilibrated at 100% A for 28 min. MS parameters were as follows: the source and de-solvation temperatures were maintained at 120 °C and 350 °C, respectively. The capillary voltage was set to 2 kV, and the cone voltage was set to 40 V. Nitrogen was used as de-solvation gas and cone gas at a flow rate of 700 L*h^−1^ and 50 L*h^−1^, respectively. The mass spectrometer was operated in full-scan HDMSE negative ionization over a mass range of 50–2000 Da. For the high energy scan function, a collision energy ramp of 20–80 eV was applied; for the low energy scan function, 5 eV was applied. Leucine-enkephalin was used as a lock-mass reference standard.

Semipolar compound identification and data analysis: LC-MS data were analyzed and processed with UNIFI (Version 1.9.4, Waters Corp., MA, USA). The putative identification of the different semipolar species was performed by comparing the accurate mass, fragmentation pattern, and ion mobility (CCS) values of theoretical structures in an in-house-generated library. The data were normalized using internal standards and sample weights.

### 2.5. Photomorphogenesis

WT and six independent ICT1 lines (1, 2, 28, 33, 478, and 8098) were used for the experiment. Seeds were exposed to 5 h of light, covered with aluminum foil, and grown at 21 °C for ten days. Hypocotyl length, cotyledon opening %, and angle were measured.

### 2.6. Abiotic Stresses

All lines were grown for two weeks and then exposed to the following conditions: 1. Salt stress: pots were irrigated with a 200 mM NaCl solution; 2. Constitutive heat (28 °C) for 3 days; 3. Intermittent high heat (44 °C) two hours a day for a week; 4. Drought: plants were irrigated with half the normal volume of water (1.5 lit/64 pots of soil); 5. UV stress: single exposure to UVb 5000 ERG for 1 min; 6. Cold stress: 4 °C during the night for a week. Untreated samples were used as controls. Plants were monitored for plant area, roundness, water content, and photosynthesis (QY max, Fv/Fm_Lss, NPQ_Lss, and RFD_Lss) using a Plant Screen^TM^ Phenotyping System (Photon Systems Instruments (PSI), Drasov, Czech Republic). Seedlings grown in MS were transferred to PSI standard pots (one seedling per pot), and imaging was performed on days 14–31 with a 3–4-day interval.

### 2.7. Statistics

All the experiments were repeated at least three times. The variance between the groups that were statistically compared was similar. The samples were analyzed using a *t*-test. We considered *p* < 0.05 to be significant.

PCA is a dimension reduction analysis that transforms the original data into a new set of uncorrelated variables (principal components (PC)) that capture the maximum variance in the data. To see how many PCs can explain the most variance, we use the scree plot. In a scree plot, the point where the eigenvalues start to level off or show a bend is often referred to as the “elbow” of the plot. The number of principal components before this bend is typically considered for retention. In our case, six PCs are the most significant ones. Thus, we have chosen the combinations between them that give the best separation between samples for visualization.

## 3. Results

### 3.1. Tolerance to Exogenous I3C in ICT1 Likely Arises from Reduced Endogenous Levels of I3C

To elucidate the mechanisms leading to I3C tolerance in ICT1, we took several approaches. To investigate if the tolerance of ICT1 mutants to I3C can be attributed to reduced uptake of extracellular I3C, we conducted I3C uptake assays using LC-MS-MS. The lines used for the assay were: ICT1, WT, *S30*^t-DNA^, *tgg1/2* (myrosinase-deficient), and *cyp79B2/B3* (deficient in indolic GLS synthesis).

**Figure 1:**
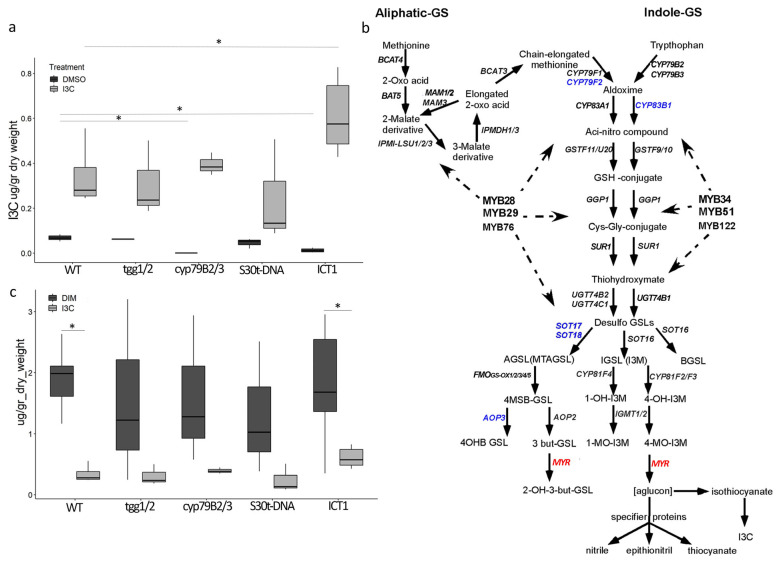
Uptake of exogenous I3C. LC-MS-MS results. (**a**) Total I3C content (mg/g dry weight) for each line. Each sample was treated with either 1 M DMSO or I3C 400 µM (in 1 M DMSO) for 2 h. (**b**) Glucosinolate pathway: genes marked in blue are down-regulated, and genes marked in red are up-regulated in ICT1. (**c**) Total DIM and I3C content following treatment with I3C (mg/g dry weight) for each line. * *p* value < 0.009 *t*-test. 3 > *n* > 6.

In control samples treated with dimethyl sulfoxide (DMSO), the measured I3C content represents I3C endogenously produced by the plant. We observed similar concentrations of endogenous I3C levels in WT and *tgg1/2* and reduced concentrations in ICT1, while no detectable I3C was observed in the *cyp79B2/B3* double mutant (Figure 1a). The similar concentrations in WT and *tgg1/2* indicate that in an undamaged plant, endogenous I3C is hydrolyzed by a non-enzymatic breakdown, consistent with previously shown myrosinase-dependent and independent I3C hydrolysis pathways [5]. The reduction in endogenous levels of I3C in ICT1 correlates with the downregulation of several glucosinolate (GLS) biosynthetic genes in ICT1 (Figure 1b). The fact that *cyp79B2/B3* and ICT1 have reduced I3C levels supports the idea that downregulation of GLS genes contributes to the lower abundance of I3C in ICT1. Interestingly, while GLS synthesis is down-regulated in ICT1, this line has increased expression of the myrosinase-encoding *tgg* genes (Figure S1). Conversely, the uptake of exogenous I3C by ICT1 mutants was found to be twice as high as in WT plants (Figure 1a). Therefore, tolerance to exogenous I3C in ICT1 is not due to defects in I3C uptake.

We next tested whether the increased degradation of I3C contributes to ICT1′s tolerance of I3C. I3C is a highly unstable metabolite that forms various oligomers (Figure S2), such as the formation of 3,3′-diindolylmethane (DIM) [18]. As depicted in Figure 1c, I3C-treated samples exhibited approximately 3-times higher levels of DIM compared to I3C alone, indicating that a significant portion of absorbed I3C was indeed degraded to DIM.

**Figure 2:**
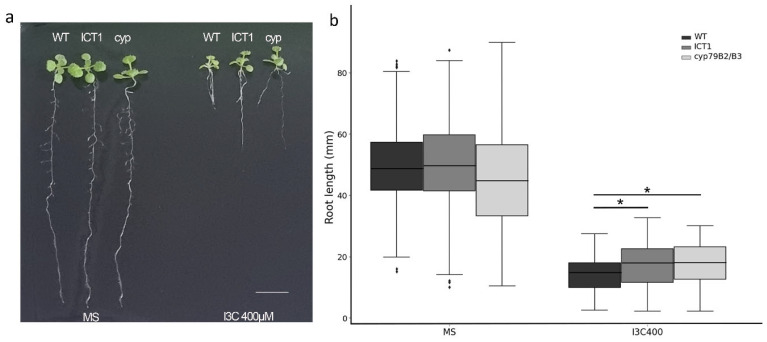
cyp79B2/B3 is tolerant of I3C. (**a**). Phenotypes of WT, ICT1, and cyp79B2/B3 on MS and I3C 400 µM after 14 days. Scale bar = 1 cm. (**b**) The average root length on MS and 400 µM I3C. *t*-test * *p* value < 0.05. 127 < *n* < 258. The dots over the whiskers are the outliers.

As endogenous I3C levels are reduced in ICT1, and as *cyp79B2/B3* (Figure 1a) contains no I3C, we thus hypothesized that *cyp79B2/B3*, like ICT1, would also be tolerant to I3C. Using the root growth assay, as seen in Figure 2, *cyp79B2/B3* is indeed tolerant to exogenous I3C. Thus, the explanation for the tolerance to exogenous I3C in both lines is that the tolerance likely arises from reduced endogenous levels of I3C.

### 3.2. ICT1 Displays Unique Metabolomic Profiles That Correlate with Known Phenotypes

**Figure 3:**
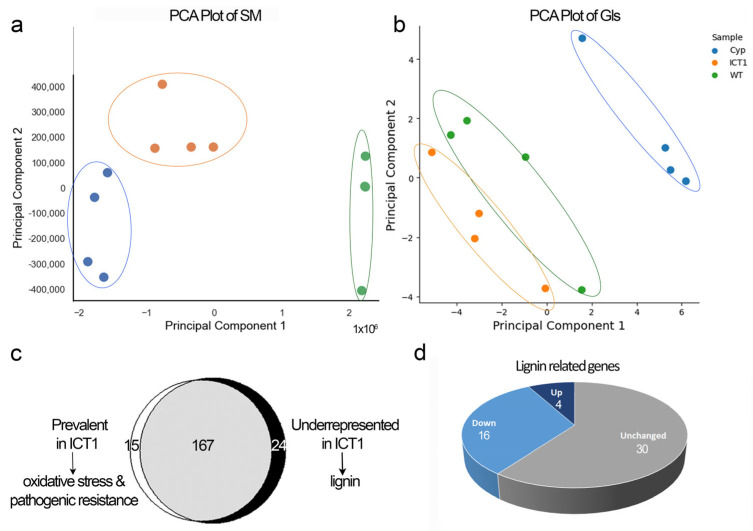
Secondary metabolites (SM) and glucosinolates (GLS) profiles. (**a**,**b**) Principal component analysis of the metabolite profiles of WT, ICT1, and *cyp*79B2/B3 for SM (**a**) and GLS (**b**). Each dot in PCA represents a biological replicate. In the case of SM, two dots in the WT sample overlap. (**c**) Out of 206 SM identified, 15 were overrepresented in ICT1, while 24 were underrepresented. In the overrepresented group, there are four SMs related to oxidative tolerance and eight related to pathogen tolerance. In the underrepresented group, the majority are lignin-related metabolites. (**d**) Out of 50 genes indicated in lignin synthesis, 16 (32%) are downregulated in ICT1 vs. WT [1]. The numbers of lignin-related genes are indicated on the pie chart.

As our previous transcriptomic study revealed differences in expression of specific metabolic pathways, we sought to determine if these differences corresponded to metabolomic changes, which led us to conduct a metabolomic profiling experiment. Specifically, we investigated the profiles of GLS metabolites (GLS) and non-GLS secondary metabolites (SM) in ICT1 compared to WT and *cyp79B2/B3*. Supplementary Table S1 presents the complete list of metabolites and abundances. The principal component analysis of this data clearly differentiates between the three lines in both SM and GLS content (Figure 3a,b). In the comparison between ICT1 and WT, we identified 39 SM that are significantly different, 24 with lower relative abundance in ICT1 and 15 with higher relative abundance (Table S1). Among the 15 prevalent SMs in ICT1, some are associated with ICT1 phenotypes (Figure 3c). Notably, our previous study demonstrated that ICT1 exhibited tolerance to both oxidative and biotic stress [1]. The tolerance to oxidative stress correlates with an increase in four specific secondary metabolites—quercitrin, linarin, citric acid, and 4-O-p-coumaroylquinic acid—each known to be effective against oxidative stress [19,20,21,22,23]. Tolerance to both bacteria and fungi correlates with five secondary metabolites—salicylic acid, kaempferol-7-O-glucoside, linarin, quercitrin, and ascorbigen, each of which shows strong antipathogen activities [23,24,25,26,27,28,29].

Furthermore, we observed three prevalent glucosinolates in ICT1: glucobrassicin, a significant indole glucosinolate serving as a precursor for active indoles; 6-hydroxyindole-3-carboxylate hexoside isomer 1; and 2-hydroxy-2-methylpropyl glucosinolate/4-hydroxybutyl glucosinolate. Indolic glucosinolates are well-known determinants for pest and disease resistance in plants [30,31,32]. Consequently, the overexpression of *S30* in ICT1 results in a higher relative abundance of metabolites associated with oxidative stress and pathogen tolerance, which correlates biochemically with the observed corresponding phenotypes.

Among the secondary metabolites with reduced content in ICT1, 62.5% are lignin related (Table S1). This reduction correlates with transcriptome results [1], which showed a down-regulation in 16 out of 50 lignin biosynthetic genes (Figure 3d).

### 3.3. I3C Treatment Impacts the Metabolic Profiles of WT More Than That of ICT1

**Figure 4:**
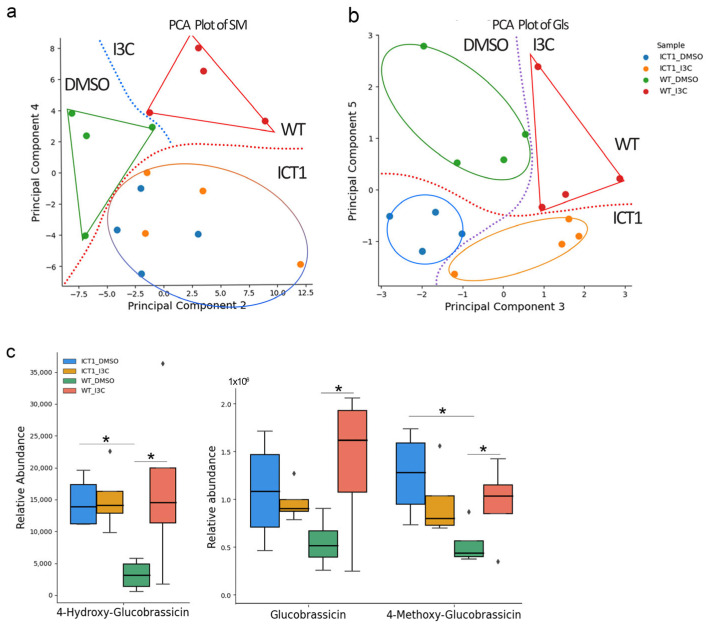
Metabolomic analysis of WT and ICT1 before and following I3C treatment. PCA plots of (**a**) secondary metabolites (SM) and (**b**) glucosinolates (GLS). The red lines differentiate between WT and ICT1. The blue and purple lines differentiate between I3C-treated and untreated. (**c**). Relative abundance of indolic GLS upstream of I3C. Following exposure to exogenous I3C, the levels of these three metabolites increase in WT while remaining constant in ICT1. * *t*-test *p* value < 0.05, *n* = 4.

We further tested the differences in metabolic profiles between WT and ICT1 before and after treatment with I3C (Table S2). Using principal component analysis, we see a clear differentiation between WT vs. ICT1 for both SM and GLS profiles, whether treated or untreated with I3C. In other words, the overexpression of 30S clearly influences the metabolic profiles of ICT1, differentiating it from WT. Following treatment of WT with I3C, the metabolic profiles of both SM and GLS are clearly separated, indicating that the I3C treatment leads to clear changes in the metabolic profiles of WT. The situation for ICT1 was, though, different. Treatment of ICT1 with I3C did not cause a significant shift in SM profile, which correlates with tolerance to I3C in this mutant (Figure 4a). In other words, just as ICT1 exhibits tolerance to I3C-induced inhibition of root growth, it also exhibits tolerance to I3C-induced changes in SM profiles.

However, the GLS profiles of ICT1 are influenced by I3C treatment (Figure 4b). However, we discovered that for three specific GLS, the I3C treatment has no effect on their levels in ICT1, while having increased levels in WT. These glucosinolates, glucobrassicin, 4-hydroxy glucobrassicin, and 4-methoxy glucobrassicin, are upstream of I3C production. The fact that they are induced by I3C treatment in WT but not in ICT1 also correlates with the tolerance in ICT1 (Figure 4c).

### 3.4. ICT1 Displays Constitutive Photomorphogenic Phenotypes

**Figure 5:**
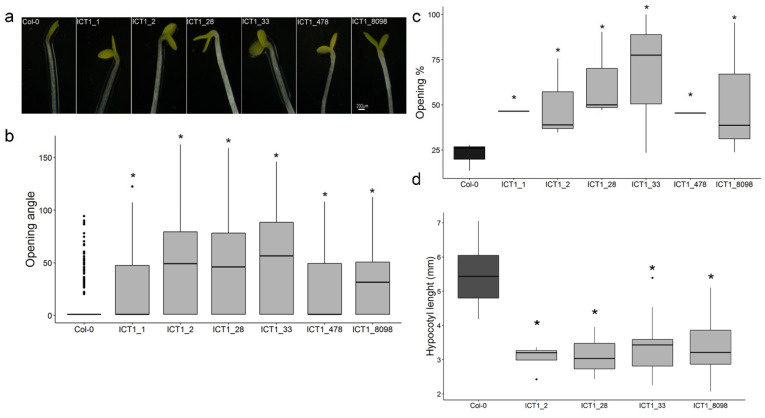
ICT1 shows a constitutive photomorphogenesic phenotype. (**a**) Representative photographs of cotyledons of 14-day-old, dark-grown WT and ICT1. Scale bar = 200 µm. (**b**–**d**) Quantitative measurements of angle of cotyledon opening (**b**), percentage of open cotyledons (**c**), and hypocotyl lengths (**d**). *n* = 55–187. * *t*-test *p* value < 0.05.

Further inspection of the transcriptome [1] revealed a group of genes related to skotomorphogenesis that are mis-regulated in ICT1, with 30 genes including Csn8 and LSH6 [33] being downregulated and 18 genes including SOB7, PCH1, CIP1, and PHOT2 [34,35,36] being upregulated (Figure S3). To test if ICT1 is affected by skotomorphogenesis, WT and six independent ICT1 lines were grown in the dark, and phenotypes were measured after 10 days. While in WT the cotyledons were closed and the hypocotyl was long, all ICT1 lines presented a constitutive photomorphogenesis (cop) phenotype of open cotyledons and short hypocotyl (Figure 5a–d). Thus, the misregulation of skotomorphogenesis-related genes in ICT1 suggests that ICT1 is defective in the regulation of photomorphogenesis.

### 3.5. ICT1 Does Not Have Wide Resistance to Abiotic Stresses

**Figure 6:**
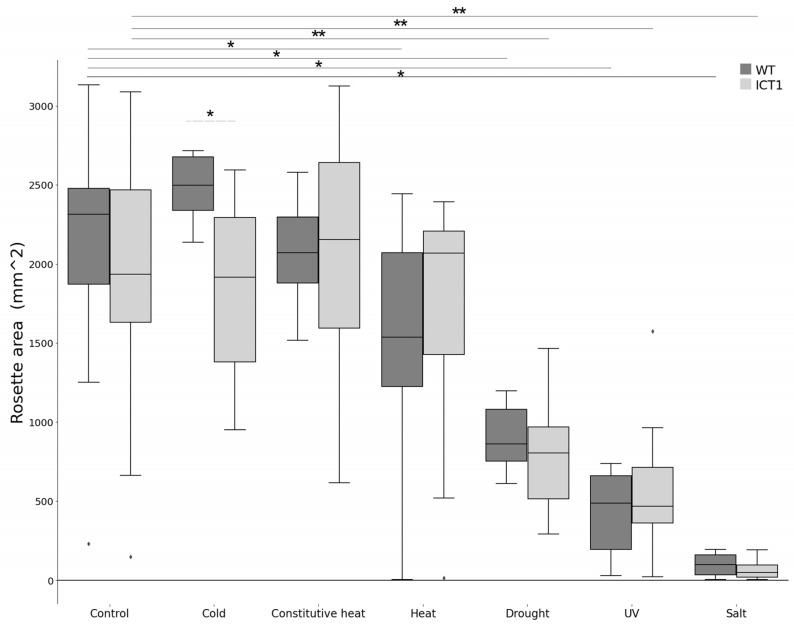
Differences in the rosette area following abiotic stresses. Rosette area following stresses: salt stress, constitutive 3 days of heat (28 °C), high heat (44 °C) 2 h a day for a week, drought, UV stress, and cold stress. Untreated samples were used as controls. *t*-test *p* value < 0.05 * WT, ** ICT1.

Since ICT1 is primed in stress response pathways and showed resistance to oxidative and biotic stress [1], we wanted to see if ICT1 has a wide range of abiotic stress resistance. To test this, six additional abiotic stresses were tested: (1) salt stress; (2) constitutive 3 days of heat; (3) high heat (44 °C) 2 h a day for a week; (4) drought; (5) UV stress; and (6) cold stress. Untreated samples were used as controls. To test if the stress caused phenotypical changes in the plants such as rosette area, leaf roundness, water content, and photosynthesis (QY max, Fv/Fm_Lss, NPQ_Lss, and RFD_Lss), we have scanned all lines by phenomics from day 14 up to day 31 with a 3–4-day interval. Not only was ICT1 not tolerant to any of these stresses, it was hypersensitive to cold treatment (Figure 6). Thus, based on these results, ICT1 did not present wide resistance to abiotic stresses, further supporting the specificity of resistance to specific biotic and abiotic challenges.

## 4. Discussion

Tolerance or resistance to specific toxic molecules can arise through several mechanisms, including but not limited to target site mutation [37], increased activity of efflux pumps that actively remove the specific molecule from inside the cell [38], reduced uptake of the molecule [39,40], and detoxification or degradation of the specific molecule [41,42,43,44]. We have shown here that tolerance to exogenously applied I3C in ICT1 is not due to defects in the uptake of exogenous I3C or increased degradation, but is likely due to reduced levels of endogenous I3C.

This conclusion is supported by our analysis of both ICT1 and the *cyp*79B2/B3 double mutant. *Cyp*79B2/B3 is deficient in GLS synthesis due to the knockout of two enzymes at the beginning of the indolic GLS pathway [17]. We showed that several genes involved in the GLS pathway are down-regulated in ICT1 (Figure 1b) (based on the transcriptome [1]). This deficiency in GLS synthesis in *cyp*79B2/B3 and the reduction in GLS synthesis in ICT1 lead to a lack or reduction of endogenous I3C levels in each line, respectively. Thus, as we found that *cyp*79B2/B3 is, like ICT1, also tolerant to exogenous I3C (Figure 2), we concluded that the tolerance to I3C in these lines results from reduced levels of endogenous I3C.

However, this reduction in I3C levels is likely not the only contributor to the I3C tolerant phenotype. As we previously showed, over-expression of *S30* in ICT1 leads to transcriptional priming, which primes the plant for the I3C treatment [1]. We further showed here that these specific transcriptional changes lead to both changes in metabolite profiles and development.

I3C is produced by the hydrolysis of indol-3-ylmethylglucosinolate (I3M-GS) in the presence of myrosinase [45]. As the glucosinolates and myrosinases are stored in different compartments, tissue rupture is necessary to bring them into contact [46]. Thus, I3C is produced mainly during pathogen attacks or mechanical damage [7]. Endogenous I3C found in the healthy, undamaged plant is a result of the non-enzymatic breakdown of I3M [5]. This is supported by similar levels of I3C in both WT and *tgg1/2* myrosinase-deficient mutants (Figure 1a). Interestingly, ICT1 overexpresses all functional myrosinase-encoding *Tgg* genes (Figure S1). This is likely a compensatory mechanism for the reduction in expression of GLS biosynthetic genes and consequent reduction in I3M levels.

We want to emphasize that the tolerance/resistance phenotypes detected for ICT1 are highly specific and correlate with specific transcriptional changes. In our original study, we showed that ICT1 also exhibited tolerance to oxidative stress and fungal and bacterial pathogens and that this tolerance was due to changes in gene expression patterns that primed the plants for response to the various treatments [1]. We showed here that these resistances are specific, as ICT1 was not tolerant to salt, heat, drought, UV, or cold stresses. Perhaps not unsurprisingly, we also did not detect transcriptional priming for these stress response pathways (based on the transcriptome [1]). Thus, just as we earlier showed that the tolerance in ICT1 is specific to I3C treatment and not a general tolerance response to all indoles/glucosinolates or other toxic molecules, the environmental tolerances identified were also specific to oxidative stress and pathogens and not to stresses in general. Thus, the overexpression of *S30* leads to specific transcriptional changes that lead to tolerance to abiotic and oxidative stresses, not a general overall stress response. It is important to notice that these specific tolerances carry with them a yield penalty (Figure S4).

The results from metabolic profiling coupled with transcriptome analysis (based on transcriptome [1]) further supported the hypothesis that overexpression of *S30* leads to changes in gene expression that influence specific metabolic pathways, which then lead to specific resistance phenotypes. Among the secondary metabolites enriched in ICT1, we notice metabolites associated with resistance to oxidative stress and pathogen resistance. These include quercitrin, linarin, citric acid, and 4-O-p-coumaroylquinic acid, each known to be effective against oxidative stress [19,20,21,22,23], and salicylic acid, kaempferol-7-O-glucoside, linarin, quercitrin, ascorbigen, and three glucosinolates, including glucobrassicin, each of which show strong antipathogen activities [23,24,25,26,27,28,29,30,31,32]. Thus, the overexpression of *S30* in ICT1 results in a higher relative abundance of metabolites associated with oxidative stress and pathogen tolerance, which provides a biochemical explanation for the observed corresponding phenotypes.

Genes related to lignin biosynthesis were also downregulated in ICT1 (Figure 3d) (based on the transcriptome [1]), and unsurprisingly, we identified a reduction in a number of lignin-related secondary metabolites (Table S1). Lignin is one of the most important secondary metabolites, which is produced by the phenylalanine/tyrosine metabolic pathway in plant cells [47,48]. However, this reduction did not correlate with any of the phenotypes tested, and indeed, a reduction in lignin would be thought to lead to an increase in pathogen or drought sensitivity. ICT1 could be a tool for further analyzing the role of lignin in plant development.

The most unexpected finding for us was that overexpression of the ribosomal gene *S30* in ICT1 affects light-mediated plant development. The misregulation of key genes (based on the transcriptome [1]), such as the down-regulation of COP9 signalosome subunit 8 (*Csn8,* also known as *Cop9*) and light-sensitive hypocotyls 6 (*LSH6*), and the up-regulation of a number of positive regulators of light signaling, including *Cop1-interating protein 1* (CIP1, [49]), photoperiodic control of hypocotyl 1 (*Pch1*), suppressor of PHYB-4 7 (*SOB7*), and phototropin 2 (PHOT2) (Figure 5), led us to test if ICT1 has a phenotype related to photomorphogenesis. Indeed, ICT1 has a constitutive photomorphogenic phenotype in the dark, including short hypocotyls and open and expanded cotyledons (Figure 6), similar, for example, to mutations in the Cop9 signalosome [33] or overexpression of positive regulators of photomorphogenesis [50]. Considering that the overexpression of *S30* interacts with I3C responses, this finding of a cop phenotype for a plant resistant to I3C is not that unexpected. Indeed, a number of studies have connected the Cop9 signalosome with hormonal as well as environmental responses [51,52], and treatment with I3C induces transcriptional changes in *Arabidopsis* similar to mutations in the CSN.

While our motivation was to further identify the mechanism of I3C tolerance in ICT1, we keep meeting the surprising findings that overexpression of a single ribosomal subunit, *S30*, triggers the misregulation of specific gene families. How this subunit mediates these responses is unclear. Indeed, the function of many individual ribosomal subunits is unclear, and in this case, we do not know if the over-expressed protein solely incorporates into the ribosome or has some mode of action in a form independent of the ribosome. Unfortunately, attempts at using antibodies or tagged proteins to answer this question were inconclusive.

Our results add to mounting evidence that differences in RP expression can impact functions unrelated to ribosome assembly or protein synthesis. For example, the constitutive expression of *L6* in rice has been linked to enhanced tolerance to salt stress (NaCl 150–200 mM) [53], while the downregulation of *S6* in cotton has resulted in reduced levels of salicylic acid and jasmonic acid, rendering cotton plants more susceptible to *V. dahliae*. On the other hand, overexpression of *S6* in *Arabidopsis thaliana* has imparted resistance to *V. dahliae* [54]. Studies employing *S. cerevisiae* also provide evidence that RP effect transcription as over-production of specific ribosomal proteins repress mutations of RNA Pol III in *S. cerevisiae* [55,56]. These and other studies indicate that individual ribosomal proteins have extra-ribosomal functions in addition to those in the translation machinery [57,58,59,60]. Future studies must elucidate the mechanism by which *S30* influences transcription.

## Data Availability

The information about the transcriptomic data can be found in [1].

## Supplementary Materials

The following supporting information can be downloaded at: https://www.mdpi.com/article/10.3390/biom14030319/s1. Figure S1: Expression of *Tgg* functional genes in ICT1 vs. WT; Figure S2: I3C hydrolysis products; Table S1: Secondary metabolites significantly different between ICT1 and WT; Table S2: A list of significantly differing metabolites following I3C treatment; Figure S3: Transcript levels of Skoto/photo morphogenesis-related genes in ICT1 vs. WT; Figure S4: Reduced yield in ICT1.s [1,5,61,62,63].

## Nomenclature

Glucosinolates (GLS); ribosomal proteins (RP); secondary metabolites (SM); relative abundance (RA).
