# Supplementary material for: Overexpression of S30 Ribosomal Protein Leads to Transcriptional and Metabolic Changes That Affect Plant Development and Responses to Stress
Source: Biomolecules. 2024 Mar 7;14(3):319. doi: 10.3390/biom14030319 (PMC10968494; doi:10.3390/biom14030319)
Supplement: Supplementary file 1 [file biomolecules-14-00319-s001.zip › biomolecules-2886946-supplementary.pdf]

Supplementary Materials

S-1. Expression of *Tgg* functional genes in ICT1 and WT. * *P value*< 0.05 (based on transcriptome [1])

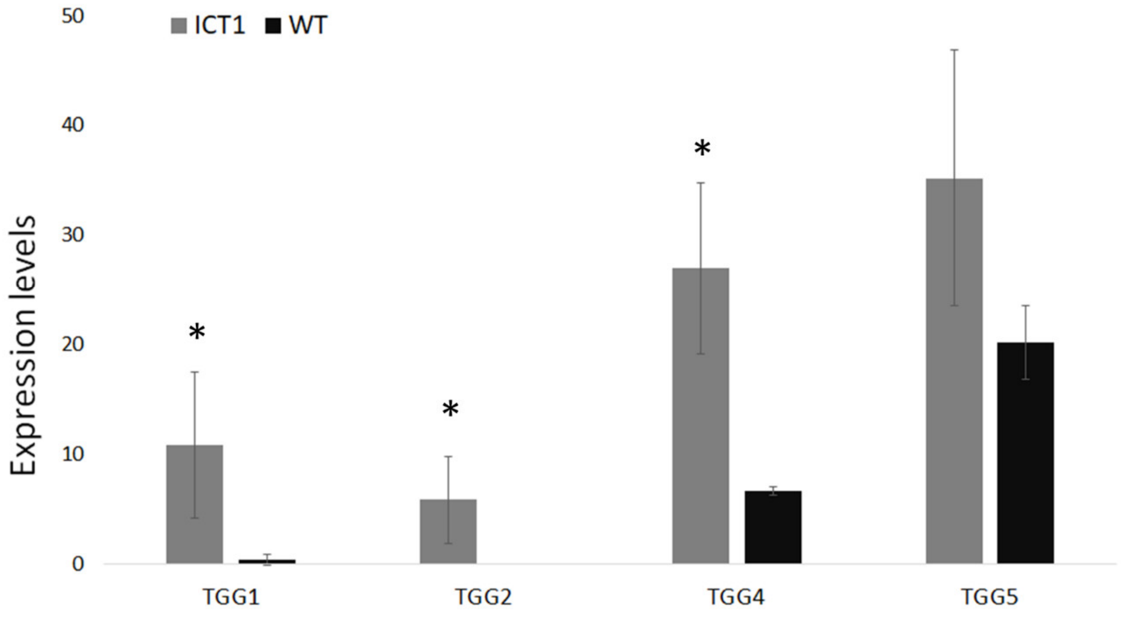

S-2. I3C hydrolysis products of I3C:

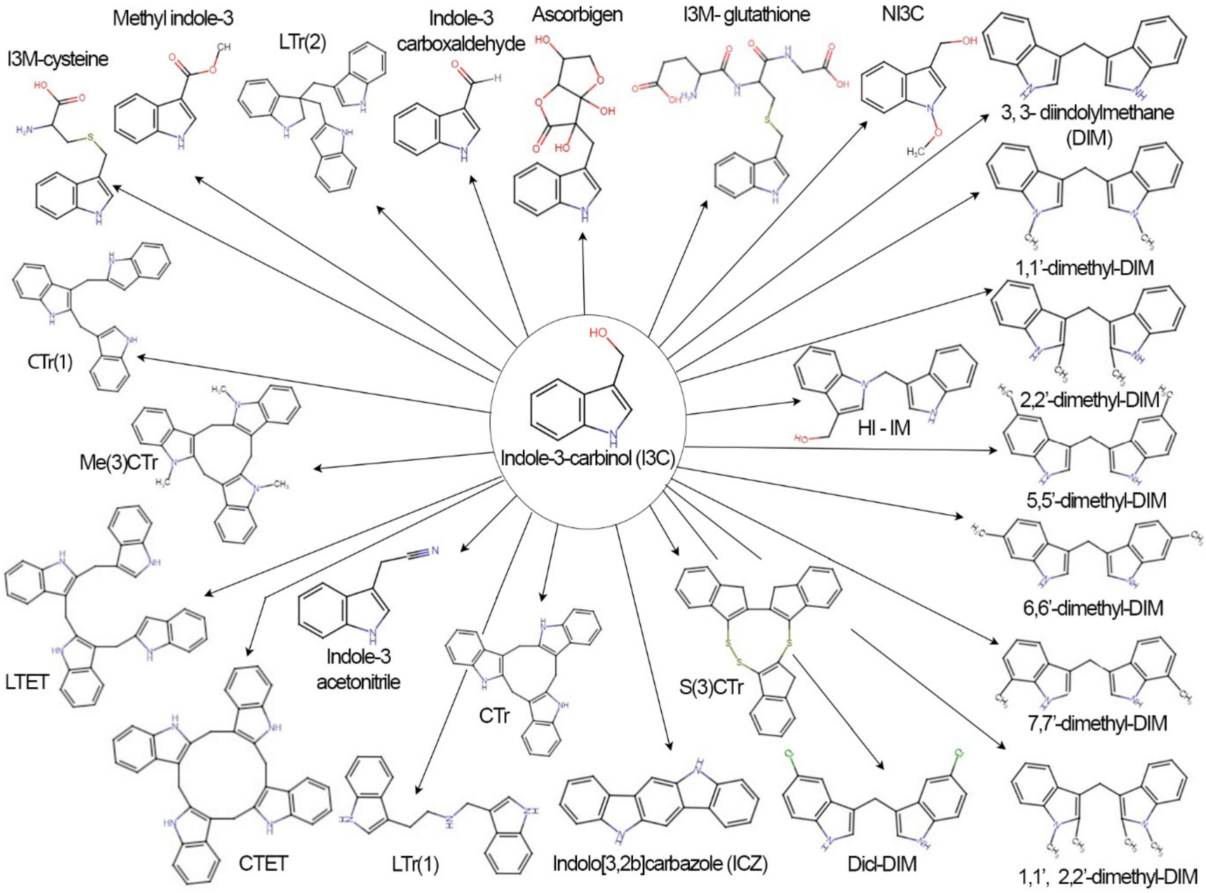

Structure of I3C and its derivates. The abbreviations used are: Ctr, 5,6,11,12,17,18-hexahydrocyclonona[1,2-b:4,5-b':7,8-b' '] triindole; andtwo tetramers, one linear (LTET) and one cyclic (CTET); DIM, 3,3'-diindolylmethane; HI-IM, 1-(3-hydroxymethyl)indolyl-3-indolylmethane; ICZ,indolo[3,2-b]carbazole; I3C, Indole-3-carbinol; LTr(1), 2-(indol-3-ylmethyl)-3,3'-diindolylmethane,Me(3)Ctr, 5,6,11,12,17,18-hexahydro-5,11,17-trimethylcy-clonona [1,2-b:4,5-b*:7,8-b**]triindole; NI3C, N-methoxyindole-3-carbinol; S(3)CTr, 9,18-dihydro-12H-[1,2,5]trithionino[3,4-b:6,7-b*:9,8-b**]triindole, indole-3-acetonitrile (Aggarwal and Ichikawa 2005, Centofanti, Buono et al. 2023). I3M cysteine, I3M ascorbate (ascorbigen), I3M glutathione, indole-3 carboxaldehyde, methyl indole 3 carboxylate (Kim, Lee et al. 2008). Indole-3 carboxaldehyde (I3A) (Katz, Nisani et al. 2018). Indole-3-carboxylic acid (I3CA).

Table S-1. Secondary metabolites significantly different between ICT1 and WT. The numbers are relative abundance (RA).

<table>
<tr><th></th><th>Secondary metabolite name</th><th>Log fold change</th><th>Ave RA value ICT1</th><th>Ave RA value WT</th><th colspan="2">Annotation</th></tr>
<tr><td colspan="7">Secondary metabolites prevalent in ICT1</td></tr>
<tr><td>1</td><td>Guaiacyl(8-O-4)ferulic acid ether hexoside Isomer 4</td><td>0.491624</td><td>202014.4</td><td>143677.5</td><td></td><td rowspan="3">Lignin</td></tr>
<tr><td>2</td><td>Guaiacyl(8-O-4)ferulic acid ether hexoside Isomer 3</td><td>0.464619</td><td>351479.7</td><td>254704.2</td><td></td></tr>
<tr><td>3</td><td>4-O-p-Coumaroylquinic acid</td><td>0.30412</td><td>330.6768</td><td>267.8272</td><td rowspan="4">Oxidative stress</td></tr>
<tr><td>4</td><td>Citric acid</td><td>0.549884</td><td>14895.38</td><td>10174.66</td><td></td></tr>
<tr><td>5</td><td>Quercitrin</td><td>2.809207</td><td>153868.4</td><td>21953.01</td><td rowspan="8">Pathogen resistance</td></tr>
<tr><td>6</td><td>Linarin</td><td>0.361421</td><td>64348.84</td><td>50088.99</td></tr>
<tr><td>7</td><td>Salicylic acid</td><td>0.233229</td><td>1352.754</td><td>1150.826</td><td></td></tr>
<tr><td>8</td><td>Kaempferol-7-O-glucoside</td><td>0.214497</td><td>5548.782</td><td>4782.2</td><td></td></tr>
<tr><td>9</td><td>Glucosyl dihydroascorbigen</td><td>0.324232</td><td>6183.618</td><td>4938.999</td><td></td></tr>
<tr><td>10</td><td>3-Indolylmethyl glucosinolate (Glucobrassicin)</td><td>0.507547</td><td>3880992</td><td>2729957</td><td></td></tr>
<tr><td>11</td><td>6-Hydroxyindole-3-carboxylate hexoside Isomer 1</td><td>0.292963</td><td>3300.207</td><td>2693.708</td><td></td></tr>
<tr><td>12</td><td>2-Hydroxy-2-methylpropyl/4-Hydroxybutyl/ glucosinolate Isomer 1</td><td>0.797718</td><td>5115.626</td><td>2942.807</td><td></td></tr>
<tr><td>13</td><td>Xanthosine</td><td>0.842999</td><td>2998.35</td><td>1671.531</td><td></td><td></td></tr>
<tr><td>14</td><td>Disinapoyl hexose Isomer 4</td><td>0.397503</td><td>56691.29</td><td>43038.39</td><td></td><td></td></tr>
<tr><td>15</td><td>N-Acetyl-L-tyrosine</td><td>0.295034</td><td>669.6142</td><td>545.7713</td><td></td><td></td></tr>
<tr><td colspan="7">Secondary metabolites underrepresented in ICT1</td></tr>
<tr><td>1</td><td>Caffeic acid</td><td>-0.40969</td><td>477.2255</td><td>633.9458</td><td></td><td rowspan="14">Lignin</td></tr>
<tr><td>2</td><td>Guaiacyl(8-O-4)Guaiacyl(8-O-4)sinapic acid ether hexoside</td><td>-0.3783</td><td>1171.457</td><td>1522.67</td><td></td></tr>
<tr><td>3</td><td>Guaiacyl(8-O-4)Syringyl(8-8)Guaiacyl hexoside Isomer 1</td><td>-0.45003</td><td>1151.112</td><td>1572.497</td><td></td></tr>
<tr><td>4</td><td>Guaiacyl(8-O-4)Syringyl(8-8)Guaiacyl hexoside Isomer 2</td><td>-0.29666</td><td>1306.541</td><td>1851.839</td><td></td></tr>
<tr><td>5</td><td>Guaiacyl(8-O-4)sinapoyl malate ether Isomer 2</td><td>-0.26905</td><td>2367.253</td><td>2852.567</td><td></td></tr>
<tr><td>6</td><td>Guaiacyl(8-O-4)Guaiacyl(8-O-4)Guaiacyl hexoside</td><td>-0.29818</td><td>2341.692</td><td>2879.317</td><td></td></tr>
<tr><td>7</td><td>Glycosmisic Acid</td><td>-0.51046</td><td>2111.947</td><td>3008.478</td><td></td></tr>
<tr><td>8</td><td>Sinapoyl dihexose Isomer 1</td><td>-0.38213</td><td>7952.119</td><td>10363.72</td><td></td></tr>
<tr><td>9</td><td>Guaiacyl(8-O-4)sinapic acid ester hexoside</td><td>-0.31674</td><td>8666.077</td><td>10793.72</td><td></td></tr>
<tr><td>10</td><td>Syringyl(8-O-4)ferulic acid ether hexoside</td><td>-0.31952</td><td>10418.15</td><td>13000.93</td><td></td></tr>
<tr><td>11</td><td>Syringin</td><td>-0.33606</td><td>178069.2</td><td>224778.3</td><td></td></tr>
<tr><td>12</td><td>Guaiacyl(8-O-4)sinapoyl malate ether Isomer 1</td><td>-0.18321</td><td>13975.89</td><td>15868.27</td><td></td></tr>
<tr><td>13</td><td>Sinapoyl malate hexose</td><td>-0.24594</td><td>14912.9</td><td>17684.65</td><td></td></tr>
<tr><td>14</td><td>Guaiacyl(8-O-4)Guaiacyl hexoside</td><td>-0.14954</td><td>21586.71</td><td>23944.39</td><td></td></tr>
</table>

15	Guaiacyl(8-O-4)lariciresinol hexoside Isomer 2	-0.50321	28385.05	34865.37		
16	Isorhamnetin 3-O-rutinoside	-0.39739	23919.9	31505.49		
17	Vomifoliol 9-O-D-glucopyranoside	-0.31948	99315.26	123933.6		
18	Kaempferol dihexoside	-0.10322	180838.2	194250		
19	1- // 2- Methylpropyl glucosinolate // Butyl glucosinolate - isomer 1	-0.26941	4113.035	4957.492		
20	4-Methylthiobutyl glucosinolate (Glucoerucin)	-0.4905	1144422	1607840		
21	Isorhamnetin trihexoside	--	0	157.9897		
22	Apodanthoside	-0.25524	486.6428	580.8252		
23	Lariciresinol	-0.4551	594.1863	814.5555		
24	Obacunone 17-glucoside (NP-021153)	-1.01048	980.4338	1975.168		

Table S-2. A list of significantly differing metabolites following I3C treatment

Secondary metabolite name	Log fold change ICT1	Ave RA ICT1 DMSO	Ave RA ICT1 I3C
2-Butenoic acid, 2-hydroxy-4-(1-methyl-1H-indol-3-yl)-4-oxo	3.86	7981.9	115861.5
6-Hydroxyindole-3-carboxylate hexoside	0.60	122686.1	186480.6
Caffeoylshikimic acid	-2.66	6591.7	1042.0
Disinapoyl hexose Isomer 2	-0.55	48381.3	32936.4
G(8-O-4)G(8-5)ferulic acid hexoside isomer 1	-0.58	2417.0	1611.6
Glucosyl dihydroascorbigen	4.50	34340.3	775067.2
Glutathione reduced	1.93	4576.9	17383.6
Hydroxyferuloyl hexose Isomer 2	-0.76	20573.1	12130.6
INDOLE-3-CARBOXYLIC ACID	--	0.0	3572.9
Naringenin	-1.59	7109.2	2368.7
Neoascorbigen	-1.01	4953.1	2463.2
Sinapoyl dihexose Isomer 2	-1.04	4552.2	2220.8
Sinapoyl malate Isomer	-0.23	474047.6	402889.7
**Secondary metabolite name**	**Log fold change WT**	**Ave RA WT DMSO**	**Ave RA WT I3C**
2-Butenoic acid, 2-hydroxy-4-(1-methyl-1H-indol-3-yl)-4-oxo	3.76	10541.1	142643.7
5-O-Feruloylquinic acid	-2.65	657.8	104.6
9,10-Dihydrohydroxy jasmonic acid sulfate	0.92	14443.5	27288.5
Coumaroylquinic acid	2.01	62.3	250.9
D-ERYTHROSE	-0.61	8008.2	5262.8
Disinapoyl hexose Isomer 2	-0.53	55850.3	38694.0
Ferulic acid	-2.02	2086.6	513.0
Feruloyl glycerol	0.67	498.7	794.4

G(8-5)feruloyl malate	0.28	13107.3	15928.9
G(8-O-4)ferulic acid ether hexoside Isomer 5	-0.61	5604.3	3672.1
G(8-O-4)feruloyl malate ether hexoside Isomer 2	0.55	4639.5	6771.1
G(8-O-4)feruloyl malate Isomer 1	0.27	25659.3	31016.2
Glucosyl dihydroascorbigen	4.71	31571.8	825143.1
GLUTAMINE	-0.59	30612.8	20321.5
INDOLE-3-CARBOXYLIC ACID	3.06	395.8	3308.9
Kaempferol-7-O-glucoside	-0.86	5581.7	3080.4
Naringenin	-1.47	10672.8	3839.5
Neoascorbigen	-0.94	3784.1	1974.7
p-coumaric acid	-1.28	3554.1	1461.1
Qn dihexoside	-0.57	24055.3	16242.2
Sinapic acid	-1.36	119576.5	46482.8
Sinapoyl dihexose Isomer 2	-1.06	5077.0	2431.5
Sinapoyl malate hexose	0.67	59483.6	94666.8
URIDINE DIPHOSPHATE Hexose	-1.21	390381.6	169030.9
Vanillic acid	-1.69	1546.2	478.0

S-3. Transcript levels of Skoto/photo morphogenesis related genes in ICT1 vs WT. The transcript levels of the displayed genes were extracted from the control light-grown transcriptome data of the two strains (Finkelshtein, Khamesa et al. 2021). Total genes tested 246, upregulated 18 (**a**), downregulated 30 (**b**).

Up regulated genes

Gene ID	Gene name	ICT1	WT
**AT1G17060**	CYP72C1	125.365	75.256667
**AT1G70800**	CAR6	222.370	82.543333
**AT2G16365**	PCH1	957.035	555.550000
**AT3G15010**	UBA2C	1136.030	765.773333
**AT3G20780**	TOP6B	289.355	174.313333
**AT3G44680**	HDA9	312.475	151.863333
**AT3G55850**	LAF3	1012.970	710.856667
**AT3G57800**	BHLH60	126.270	63.100000

Down regulated genes

Gene ID	Gene name	ICT1	WT
**AT1G07090**	LSH6	109.460	241.066667
**AT1G10370**	GSTU17	41.725	166.663333
**AT1G29060**	ATSFT12	150.330	241.233333
**AT1G30210**	TCP24	190.725	349.576667
**AT1G70990**	EXT33	59.770	189.266667
**AT1G74660**	MIF1	314.200	518.413333
**AT2G42080**	BIL2	136.015	191.420000
**AT2G42610**	LSH10	10.985	63.713333

**AT4G03400**	DFL2	160.325	107.736667	**AT2G43950**	OEP37	234.575	332.723333
**AT4G12500**	lipid-transfer protein	50.610	2.250000	**AT2G45430**	AHL22	1067.495	1470.153333
**AT4G12510**	AZI5	187.765	60.440000	**AT2G46340**	SPA1	571.915	835.683333
**AT4G16180**	transmembrane protein	1116.750	817.070000	**AT2G46370**	JAR1	1036.255	1661.543333
**AT4G21380**	SD18	28.800	9.970000	**AT2G46970**	PIL1	15.905	35.963333
**AT5G03500**	MED7B	475.615	227.543333	**AT3G01470**	HAT5	1732.355	2966.043333
**AT5G23060**	CAS	68.060	26.680000	**AT3G15030**	TCP4	68.460	172.363333
**AT5G24630**	BIN4	356.860	224.113333	**AT3G16010**	MISF68	46.310	90.363333
**AT5G41790**	CIP1	998.885	689.436667	**AT3G16570**	RALF23	118.150	183.970000
**AT5G58140**	PHOT2	183.680	114.373333	**AT3G16920**	CTL2	126.770	213.403333
				**AT4G14110**	CSN8	665.370	885.686667
				**AT4G18610**	LSH9	151.380	278.323333
				**AT4G33495**	RPD1	134.740	244.863333
				**AT4G37470**	KAI2	382.075	619.883333
				**AT4G38740**	CYP18-3	4370.920	8121.886667
				**AT5G02580**	RPGE1	120.260	206.736667
				**AT5G25220**	KNAT3	226.400	347.570000
				**AT5G58500**	LSH5	29.800	81.353333
				**AT5G59920**	ULI3	23.340	82.626667
				**AT5G62000**	ARF2	1572.590	2103.333333
				**AT5G62430**	CDF1	13.895	48.276667
				**AT5G63980**	SAL1	602.200	857.480000

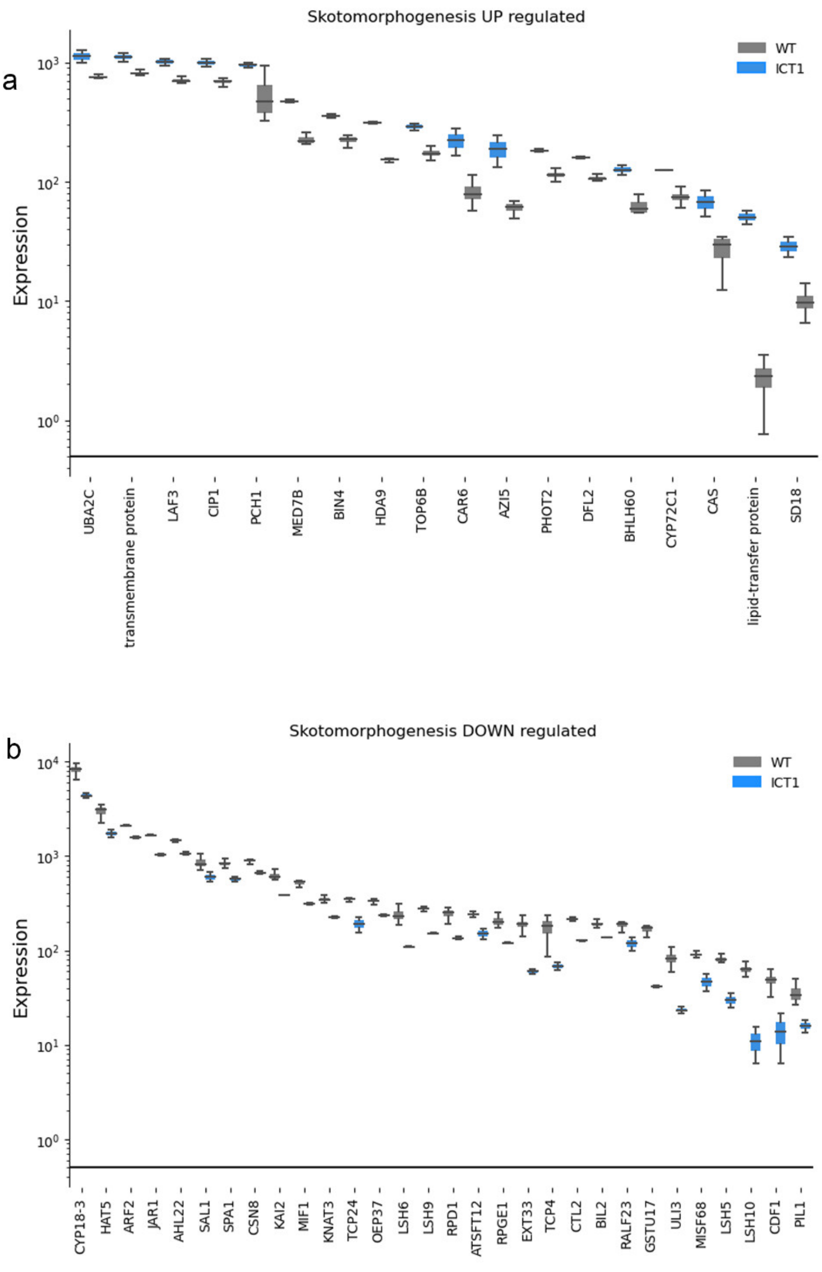

S-3. Reduced yield in ICT1. * TTEST *P.value* <0.05.

In our previous study, we remarked that overexpression of S30 has potential agronomic value due to its increased tolerance to pathogens and oxidative stress. To further examine the feasibility of such an approach, we examined if this resistance is accompanied by any yield cost that might offset the potential gains. ICT1 and WT lines were grown in optimal conditions until full ripening. Seeds were collected separately and weighed. The weight was transformed to number of seeds as seen in S4 with aid of calibration curve. ICT1 has a

reduction in yield as measured in seeds per/plant, indicating that overexpression of S30 carries with it a yield penalty.

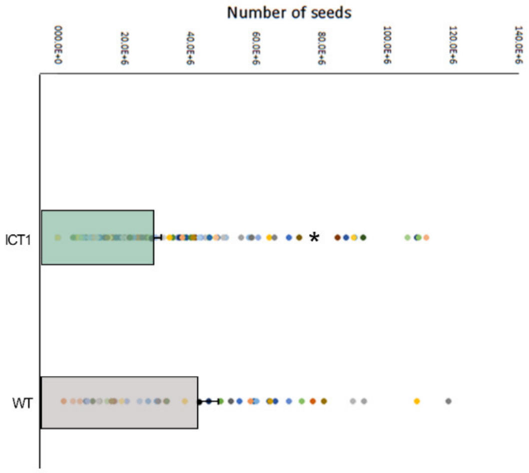

References

- Aggarwal, B. B. and H. Ichikawa (2005). "Molecular Targets and Anticancer Potential of Indole-3-Carbinol and Its Derivatives." Cell Cycle **4**(9): 1201-1215.
- Centofanti, F., A. Buono, M. Verboni, C. Tomino, S. Lucarini, A. Duranti, P. P. Pandolfi and G. Novelli (2023). "Synthetic Methodologies and Therapeutic Potential of Indole-3-Carbinol (I3C) and Its Derivatives." Pharmaceuticals (Basel) **16**(2).
- Finkelshtein, A., H. Khamesa, L. A. Tuan, M. Rabanim and D. A. Chamovitz (2021). "Overexpression of the ribosomal S30 subunit leads to indole-3-carbinol tolerance in Arabidopsis thaliana." The Plant Journal **105**(3): 668-677.
- Katz, E., S. Nisani and D. A. Chamovitz (2018). "Indole-3-carbinol: a plant hormone combatting cancer." F1000Research **7**: F1000 Faculty Rev-1689.
- Kim, J. H., B. W. Lee, F. C. Schroeder and G. Jander (2008). "Identification of indole glucosinolate breakdown products with antifeedant effects on Myzus persicae (green peach aphid)." The Plant Journal **54**(6): 1015-1026.
